# Can Procalcitonin Reduce Unnecessary Voiding Cystoureterography in Children with First Febrile Urinary Tract Infection?

**Published:** 2014-07-19

**Authors:** Aliasghar Halimi-asl, Amir Hossein Hosseini, Pooneh Nabavizadeh

**Affiliations:** Department of Pediatrics, Shahid Beheshti University of Medical Sciences, Tehran, Iran

**Keywords:** Urinary Tract Infection, Procalcitonin Levels, Vesicoureteral Reflux, Voiding Cystourethrogram

## Abstract

***Objective:*** Recently, new predictors of vesicoureteral reflux (VUR) in children with a first febrile UTI such as Procalcitonin (PCT) were introduced as selective approaches for cystography. This study wants to show the capability of PCT in predicting presence of VUR at the first febrile UTI in children.

***Methods:*** Patients between 1 month and 15 years of age with febrile UTI were included in this prospective study. PCT values were measured through a semi-quantitative method in four grades comprising values less than 0.5, 0.5-2.0, 2.0-10.0 and above 10.0 ng/ml. The independence of PCT levels in predicting VUR were assessed after adjustment for all potential confounders using a logistic-regression model.

***Findings:*** A total of 68 patients, 54 (79.4%) girls and 14 (20.6%) boys were evaluated. PCT level demonstrated a significant difference between patients with positive VUR and those with negative VUR (*P*=0.012). To calculate the independent factors that may predict the presence of VUR, all included variables were adjusted for age and sex. Results of logistic regression showed that a PCT level between 2.0 and 10.0 ng/mL could independently predict presence of VUR (Odds ratio=6.11, CI 95%= 1.22-30.77, *P*=0.03).

***Conclusion:*** Our finding in this study showed that readily available semi-quantitative measures for PCT are feasible for detecting patients with VUR. We suggest that in semi-quantitative measurements of PCT, levels between 2.0 and 10.0 ng/ml could be an independent predictor of positive VUR.

## Introduction

Urinary tract infections (UTIs) are amongst the most prevalent infections of the childhood. It has been estimated that as much as 7% of girls and 2% of boys will experience at least one episode of UTI before 6 years of age^[^^1^^]^. Vesicoureteral reflux (VUR), as a retrograde flow of urine from the bladder to the kidneys, is a common complication after UTI. So far, it has been stated that a first episode of febrile UTI leads to the identification of VUR in as much as 30% of the patients^[^^2^^]^. VUR is believed to play role as a risk factor for recurrent pyelonephritis and renal scarring. Recurrent pyelonephritis, in turn, results in progressive renal failure, hypertension and proteinuria^[^^2^^]^. Given the importance of VUR in producing long term complications, early diagnosis is of great importance. Hence, systematic voiding cystourethrography (VCUG) has been recommended after the first febrile UTI in children^[^^3^^,^^4^^]^. However, for 60% to 80% of the children, VCUG could be reported as normal^[^^4^^]^. Moreover, there are several limitations to VCUG. This procedure is irradiating, painful, and expensive and is shown to be associated with an increased risk for iatrogenic UTI^[^^5^^]^. Thus, it seems the use of VCUG should be confined to patients with a greater risk of VUR. To propose additional diagnostic modalities for risk stratification of the patients and select proper population for the VCUG study, renal ultrasonography and a highly sensitive VUR risk score have been studied. The two techniques showed to have a low sensitivity and reproducibility^[^^6^^-^^10^^]^. 

 Recently, new predictors of VUR in children with a first febrile UTI such as Procalcitonin (PCT) were introduced as selective approaches for cystography. PCT as a specific marker for acute bacterial infection, has been found to be correlated with the severity and mortality of various infectious diseases^[^^11^^]^. So far, it has been stated that PCT is a reliable marker for predicting renal parenchymal inflammation in a first occurrence of UTI in infants and children^[^^12^^]^. It seems higher PCT levels show a positive correlation with subsequent permanent renal damage^[^^13^^]^. This study wants to show the capability of PCT in predicting presence of VUR at the first febrile UTI in children.

## Subjects and Methods

We conducted a prospective hospital-based cohort study in the Department of Pediatrics of Shohadaye Tajrish teaching hospital and Mofid Children’s Hospital in Tehran, Iran. Patients with their first febrile UTI admitted September 2009 to March 2012 were enrolled in the study.

 Inclusion criteria required all patients to be between 1 month and 15 years of age with first febrile UTI. Patients who received systemic antibiotics before presentation to the medical centers, those with a previously confirmed uropathy and children with a history of previous UTI were excluded from the study. Febrile UTI group was defined as having a core temperature 38°C associated with a positive bacterial urine monoculture and/or pyelonephritic change revealed on technetium-99m dimer-captosuccinic acid (DMSA) scintigraphy. Since the DMSA scan was obtained within 7 days of admission, there existed patients whose urine cultures were negative (given the use of appropriate antibiotic regimen), but their DMSA scan was in accordance with diagnosis of febrile UTI. Urine specimens were obtained mainly by midstream clean-void catching. Urethral catheterization and suprapubic aspiration were used when the midstream catching was not feasible. DMSA studies with a gamma camera (Millenium MPR; General Electric, Milwaukee, Wisconsin) were performed 4 hours after the intravenous injection of an age-adjusted dose of technetium-DMSA (range 40-100 MBq). Renal pathologic findings were defined by scintigraphy as focal or multifocal perfusion defects or a diffuse decrease in, or absence of, DMSA uptake. On admission and before initiating antibiotic treatment, all patients underwent clinical evaluation and laboratory investigations. In case of clinical data, core temperature and the presence of vomiting, diarrhea, or decreased oral intake before admission were recorded. Laboratory investigations included leukocyte count, ESR, CRP, and PCT measurement through a semi quantitative method. To determine the PCT level, a sample of 2 milliliters blood was centrifuged, serum separated and assessed using a rapid, semi quantitative immunochromatographic test (Brahms Diagnostica, Hennigsdorf, Berlin, Germany). 

 Renal ultrasonography was performed in all patients. After appropriate antibiotic treatment and achieving negative urine culture, VCUG was done in radiology department. For reducing radiation exposure to ovaries, we performed direct radionuclide cystography (DRNC) instead of VCUG in girls with normal renal sonogram.

 In this study, PCT values were measured in four grades. These grades comprised values less than 0.5, 0.5-2.0, 2.0-10.0 and above 10.0 ng/mL. Univariate analysis was conducted using the chi square or Fisher’s exact test to evaluate the relationship between the PCT levels and presence of VUR. Moreover, the relationship between the patients’ demographics and occurrence of VUR was established to be inserted in the higher level analysis. The independence of PCT levels in predicting VUR were assessed after adjustment for all potential confounders using a logistic-regression model.

## Findings

A total of 68 patients, 54 girls (79.4%) and 14 boys (20.6%), met the inclusion criteria and were evaluated in the study. Of these children, 25 (36.8%) were between 1 and 12 months, 33 (48.5%) older than 12 months and younger than 5 years of age. Five (14.7 %) patients were between 6 and 15 years of age.

**Table 1:** Patients’ demographic, clinical and laboratory characteristics

**Variables**	**Positive VUR (n=27)** **n (%)**	**Negative VUR (n=41)** **n (%)**	***P. *** **value**
**Median age (months)**	4	5	1
**Female gender**	21 (77.8)	33 (80.5)	1
**Fever**	25 (92.6)	39 (95.1)	1
**Systemic symptoms**	24 (88.9)	37 (90.2)	1
**Urinary symptoms**	16 (59.3)	26 (63.4)	0.8
**WBC>15000**	17 (63)	20 (48.8)	0.3
**CRP>6**	16 (59.3)	33 (80.5)	0.1
**Positive ESR**	22 (81.5)	33 (80.5)	1
**Renal scar on DMSA scan**	26 (100)	37 (94.9)	0.5
**Hydronephrosis in sonogram**	4 (14.8)	3 (7.3)	0.4
**PCT levels <0.5** ** 0.5-2** ** 2-10** ** >10**	5 (18.5) 7 (25.9) 13 (48.1) 2 (7.4)	11 (26.8) 18 (43.9) 5 (12.2) 7 (17.1)	0.01

CRP: C-Reactive protein; WBC: White blood cells; ESR: Erytocyte sedimentation rate; DMSA: dimercaptosuccinic acid

PCT levels, as measured by semi quantitative method, showed that 16 (23.5%) patients were in <0.5 ng/ml group, 25 (36.7%) in 0.5-2.0 ng/ml, 18 (26.5%) in 2.0-10.0 ng/ml and 9 (14.7%) in >10.0 ng/ml group. No adverse event was reported during PCT measurement and cystography.

 In first level analysis, PCT level demonstrated a significant difference between patients with positive VUR and those with negative VUR (*P*=0.012). Table 1 summarizes patients’ demographics, important clinical and laboratory findings and their distribution between the two study groups (patients with positive VUR and those who had no VUR). 

 For the second level analysis, variables that showed a statistically significant difference between the VUR positive and VUR negative groups were entered in the logistic model. To calculate the independent factors that may predict the presence of VUR, all included variables were adjusted for age and sex. Results of logistic regression showed that a PCT level between 2.0 and 10.0 ng/mL could independently predict presence of VUR (Odds ratio=6.11, CI 95%=1.22-30.77, *P*=0.028). This is while other PCT levels and the other included variables in the model could not predict the presence of VUR. These results are summarized in Table 2.

 To define best cutoff in terms of sensitivity and specificity three different cutoff points of 0.5 ng/ml, 2 ng/ml and 10 ng/ml were evaluated. The sensitivity and specificity of VUR prediction using different cutoff points are shown in Table 3.

## Discussion

In the present study, it is shown that a PCT level between 2.0 and 10.0 ng/ml can be an independent predictor for the presence of VUR. This finding has important clinical implications and might facilitate an earlier evaluation with VCUG.

**Table 2:** Logistic regression results

**Variables**	**Wald**	**Odds Ratio**	**Confidence Interval (95%)**		***P*** **-value**
			**Lower**	**Upper**	
**CRP >6**	3.75	-	-	-	0.05
**Sex**	0.32	-	-	-	0.6
**Age**	0.048	-	-	-	0.8
**PCT**	10.36	-	-	-	0.02
**0.5<PCT<2.0**	0.22	-	-	-	0.6
**2.0<PCT<10**	4.83	6.11	1.22	30.77	0.03*
**PCT >10**	0.275	-	-	-	0.6

CRP: C-Reactive protein; PCT: Procalcitonin

* PCT levels between 2.0 and 10.0 ng/ml can independently predict presence of Vesicoureteral reflux

**Table 3:** Sensitivity and specificity of Procalcitonin cutoff points

**Procalcitonin cutoff value**	**Sensitivity**	**Specificity**
0.5	77%	26%
2	51%	70%
10	8%	82%

 Presence of VUR may predispose children to frequent UTIs and subsequent renal impairments and hence early diagnosis of this disease is of great importance. Because of the expenses and technical difficulties in VCUG and the imprecision of ultrasonography for the detection of VUR, previous reports tried to introduce a new and more convenient diagnostic tool for early detection of VUR. Imaging techniques could not be served as successful alternatives and hence biochemical markers are the spotlight for the research for this dilemma. 

 In 1993, for the first time, Assicot and colleagues proposed PCT, a precursor of calcitonin, as an infection marker^[^^14^^]^. This marker is a sensitive and specific indicator of bacterial infections^[^^12^^]^ and is used as a reliable and sensitive predictor of high-grade VUR^[^^10^^,^^15^^]^. Similarly, in our study the results showed that the PCT levels between 2.0 and 10.0 ng/mL could independently predict presence of VUR with a high odds ratio (odds=6.11). In line with our findings, there were studies that confirmed the ability of PCT in prediction of VUR. One important issue that should be addressed here is the cutoff value for PCT to predict the presence of VUR. Thus far, it has been stated that the relation between PCT levels and VUR is significantly stronger as VUR grade increases^[^^10^^]^. This is while most of the previous reports indicated that only PCT levels more than 0.5 ng/ml could be served as independent predictors of presence of VUR. Recently, a meta-analysis by Leroy and colleagues has reviewed 12 high quality studies and concluded that the sensitivity of PCT >0.5 ng/mL was 83% (95% CI, 71-91) with 43% specificity rate (95% CI, 38-47)^[^^10^^]^. Unlike this dichotomized approach, we believe that one of the superiorities of the current study is reporting an interval for PCT levels rather than a single cutoff.

 Leroy et al previously derived a clinical decision rule for predicting VUR using serum PCT levels and the presence of ureteral dilatation on renal sonogram^[^^16^^]^. This rule’s reproducibility was then tested and validated in a separate study^[^^17^^]^. However, in both studies, unlike the present study, PCT was measured through quantitative method. Quantitative method, although provides a more precise estimation, but in comparison to semi-quantitative method, might not be available in some hospitals. On the other hand, the physician can easily do the semi-quantitative method at bedside.

 Previous reports have shown that normal level of PCT in healthy subjects is less than 0.1 ng/mL while this range reaches as high as 1000 ng/ml in patients with evident infections^[^^14^^]^.

 There exist studies that focus on the independent and significant role of PCT in predicting VUR. In the present study, we not only showed that 2.0<PCT<10.0 is the only independent factor for the presence of VUR, we have tested three different thresholds to identify best diagnostic cutoff regarding sensitivity and specificity. As it is shown in Table 3, a cutoff of 2.0 yields a justifiable diagnostic accuracy (sensitivity of 51% and specificity of 70%). This finding is in line with our logistic regression result. On the other hand, previous reports found that the best threshold for the fewest possible misdiagnoses is 0.5 ng/mL^[^^10^^,^^15^^]^. It has been revealed that discriminating power of a serum PCT level >0.5 ng/ml offers a sensitivity rate of 83% (95% CI, 71-91) and a specificity rate of 43% (95% CI, 38-47) for high-grade VUR^[^^10^^]^. Although these results are different from our findings, semi-quantitative measurement of PCT in our study confines validity of our results in reporting best cutoff for producing optimum sensitivity and specificity.

 There are certain limitations to this study. Small sample size could be named as the main confining factor of the present study. Hence additional studies with larger sample size should be conducted. Moreover, this study used PCT as a categorical variable. This measurement may result in missing certain values, although as stated earlier, we believe the feasibility of semi-quantitative measurement of the PCT may justify this approach. 

 Identical method of urine sampling for all cases In future studies might increase the inter validity of the study, though, using three different samplings, as used in the current study, can significantly increase the external validity of the results.

## Conclusion

Our findings, which were in concert with previous reports, showed that PCT can be successfully used as an indicator of VUR in febrile UTIs. Moreover we found that readily available semi-quantitative measures for PCT are also feasible for detecting patients with VUR. We also suggest that if semi-quantitative means are used for measurement of PCT, levels between 2.0 and 10.0 ng/ml could be an independent predictor of positive VUR.
